# Investigating Receptivity and Affect Using Machine Learning: Ecological Momentary Assessment and Wearable Sensing Study

**DOI:** 10.2196/46347

**Published:** 2024-02-07

**Authors:** Zachary D King, Han Yu, Thomas Vaessen, Inez Myin-Germeys, Akane Sano

**Affiliations:** 1 Department of Electrical and Computer Engineering Rice University Houston, TX United States; 2 Center For Contextual Psychiatry Katholieke Universiteit Leuven Leuven Belgium; 3 Department of Psychology, Health & Technology University of Twente Enschede Netherlands

**Keywords:** mobile health, mHealth, affect inference, study design, ecological momentary assessment, EMA, just-in-time adaptive interventions, JITAIs, receptivity, mobile phone

## Abstract

**Background:**

As mobile health (mHealth) studies become increasingly productive owing to the advancements in wearable and mobile sensor technology, our ability to monitor and model human behavior will be constrained by participant receptivity. Many health constructs are dependent on subjective responses, and without such responses, researchers are left with little to no ground truth to accompany our ever-growing biobehavioral data. This issue can significantly impact the quality of a study, particularly for populations known to exhibit lower compliance rates. To address this challenge, researchers have proposed innovative approaches that use machine learning (ML) and sensor data to modify the timing and delivery of surveys. However, an overarching concern is the potential introduction of biases or unintended influences on participants’ responses when implementing new survey delivery methods.

**Objective:**

This study aims to demonstrate the potential impact of an ML-based ecological momentary assessment (EMA) delivery system (using receptivity as the predictor variable) on the participants’ reported emotional state. We examine the factors that affect participants’ receptivity to EMAs in a 10-day wearable and EMA–based emotional state–sensing mHealth study. We study the physiological relationships indicative of receptivity and affect while also analyzing the interaction between the 2 constructs.

**Methods:**

We collected data from 45 healthy participants wearing 2 devices measuring electrodermal activity, accelerometer, electrocardiography, and skin temperature while answering 10 EMAs daily, containing questions about perceived mood. Owing to the nature of our constructs, we can only obtain ground truth measures for both affect and receptivity during responses. Therefore, we used unsupervised and supervised ML methods to infer affect when a participant did not respond. Our unsupervised method used *k*-means clustering to determine the relationship between physiology and receptivity and then inferred the emotional state during nonresponses. For the supervised learning method, we primarily used random forest and neural networks to predict the affect of unlabeled data points as well as receptivity.

**Results:**

Our findings showed that using a receptivity model to trigger EMAs decreased the reported negative affect by >3 points or 0.29 SDs in our self-reported affect measure, scored between 13 and 91. The findings also showed a bimodal distribution of our predicted affect during nonresponses. This indicates that this system initiates EMAs more commonly during states of higher positive emotions.

**Conclusions:**

Our results showed a clear relationship between affect and receptivity. This relationship can affect the efficacy of an mHealth study, particularly those that use an ML algorithm to trigger EMAs. Therefore, we propose that future work should focus on a smart trigger that promotes EMA receptivity without influencing affect during sampled time points.

## Introduction

### User Engagement in Mobile Health Systems

Mobile health (mHealth) technologies continue to grow within the health care sector and are imperative for precision medicine initiatives. mHealth can provide beneficial interactions between health care providers and patients outside clinical settings. An engaged and responsive user base in any mHealth system is vital for maximizing the knowledge that researchers and providers acquire. Mental health research mainly depends on active users because investigators rely on participant survey responses to establish ground truth. Researchers can only adequately interpret the relationships between physiology and psychological state with a population that is compliant with sensors and surveys. Evaluating a health construct is only possible with highly receptive participants in mHealth studies.

Here, we discuss 2 forms of interaction between participants and mHealth systems: ecological momentary assessments (EMAs) and just-in-time interventions (JITIs). EMAs gather in situ data from users in real time. EMAs are commonly used in mHealth studies, as they allow researchers to prompt participants regularly throughout the day [1]. In the case of mHealth studies focusing on psychological states, EMAs enable users to report their momentary symptoms or context in a natural environment, often using smartphones, because of their accessibility. JITI is a method that allows investigators to send interventions as needed. The just-in-time adaptive intervention (JITAI) uses incoming information (physiological, contextual, or psychological markers) as context to determine when an intervention is required [2]. Researchers have been working on enhancing the efficiency of these interactions. As mentioned previously, this effort is crucial because ineffective interactions in an mHealth study can have significant effects on outcomes. Failing to collect EMA responses may impede researchers’ ability to identify real-world measures of health behaviors, and without participants receiving or engaging in JITIs, researchers may find it challenging to measure the efficacy of the intervention.

### Improving EMA Receptivity

To enhance compliance with EMAs and JITIs, it is imperative to gain a comprehensive understanding of the factors that influence participant adherence. Ho and Intille [3] described 11 factors that influence a person’s interruptability (willingness to follow through if notified or interrupted). These factors encompass contextual aspects, such as social engagement, ongoing activities, future schedule, and emotional state, as well as message-related attributes, including frequency, complexity, modality, and utility.

Currently, many researchers have reduced interruptability by altering message-related attributes, often involving strategies such as reducing the complexity or frequency of an EMA or increasing the incentives for a response [4,5]. Reducing the size of the instrument relieves some of the burden associated with answering an EMA [6]. This is done by excluding redundant questions or by choosing a less complex instrument. The Perceived Stress Scale [7] was initially a 14-item question set. However, after some statistical analysis, researchers found that a 10-item instrument was sufficient for measuring stress. Another factor affecting receptivity is the frequency at which users are sampled. In 2 separate reviews, researchers demonstrated conflicting findings regarding the effects of frequency on EMA compliance [8,9]. These conflicting results can be attributed to the author's focus on differing populations and the many other factors that play a role in EMA compliance. The third method for improving receptivity rates is to increase the incentives based on EMA compliance. However, this method can be costly and seen as exploitative, especially when dealing with susceptible populations.

An emerging method for improving receptivity rates is the use of machine learning (ML). This can be achieved by using wearable data to predict the likelihood of a response, which can help deliver EMAs that mitigate interruptability. Mishra et al [10] used ML models built from previously collected data to improve the receptivity of a JITAI by contacting users at points where they are more likely to be receptive. The study showed a difference of >38% in receptivity rates between an ML-based static model (using previously collected data) and a control model (using a set schedule) to distribute EMAs. Mishra et al [11] built a model for predicting the optimal time to send an EMA. Their results demonstrated that a model built from contextual cues such as activity, audio, conversation, and location could significantly outperform a baseline model (prediction based on the proportion of responded EMAs). Researchers have also shown that contextual cues, including location [12,13], personality traits [14,15], physical activity [14,16], and time of day [17], influence participants’ willingness to respond to regular surveys. Together, these methods can predict and respond to the unobserved contextual aspects of an interruption, thus offering a more holistic approach to addressing participant engagement. However, a system that reacts to these contextual aspects may have unintentional effects on the response of the user. For instance, emotional state is an underlying factor that affects receptivity. A model designed to initiate EMAs when a participant is most likely to respond favors prompting users when experiencing positive emotions. Consequently, this approach could influence the reported emotional state during each prompt, potentially making it challenging to collect subjective responses during negative emotions. Understanding the influence of ML-based EMA triggers on these underlying receptivity factors allows us to incorporate additional variables into an algorithm. Integrating predicted affect into the decision-making of an ML-based EMA trigger will ensure that participants receive prompts across a broad spectrum of emotions.

### Relationship Between Affect and EMA Receptivity

Clark and Watson [18] described how positive and negative affect (NA) can influence participation in activities of daily living. Their results show differences in the expected mean across many social activities, with reported positive affect (PA) having more significance in differentiating the 2 groups. Similarly, research has also demonstrated a negative relationship between students’ emotional state and academic achievement [19,20]. Although none of these studies demonstrate the relationship between affect and EMA receptivity during mHealth studies, they all demonstrate the effect of emotional state on a participant’s general ability to engage in normal activities of daily living.

Several authors have examined the effect of emotional state on EMA adherence by using the preceding response as a gauge of affect during instances of nonresponse. Murray et al [21] conducted a study (N=261) demonstrating that NA and stress reduce the chance of a response during the next prompt. Other researchers have expanded on this by examining various contextual cues within an EMA that precede instances of nonresponse. The authors found that variables such as medication use, activity, battery life, and being away from home negatively impacted the compliance of the following EMA [22,23]. This work contributes to understanding how affect can influence participants’ response behavior but falls short of providing real-time explanations for the absence of responses. Alternatively, real-time explanations for receptivity can be derived through passive sensing and ML. Leveraging these explanations allows for delivering EMAs at moments of heightened receptivity, guided by current contextual and physiological factors.

### Objectives and Hypothesis

This study aims to analyze the relationship between participant EMA receptivity and affect in a 10-day wearable and EMA–based affect-sensing study (N=45). We hypothesize that a relationship exists between EMA receptivity and affect in mental health–related mHealth studies. We can establish the relationship between emotions when participants respond. However, to investigate this connection during nonresponses, we need to infer affect when a participant fails to provide a response. Therefore, we implemented ML models for identifying receptive time points and predicting emotional states. This allowed us to determine whether there was a statistically significant difference in emotions between responses and nonresponses. If this relationship exists and the likelihood of a response is dependent on emotional state, it would bias the outcome of an ML-based EMA delivery mechanism.

## Methods

### Ethical Considerations

Ethics approval was granted by the *Sociaal-Maatschappelijke Etische Commissie* of Katholieke Universitei Leuven (G-2018 09 1339) [24]. Informed consents were obtained from the participants. All data was de-identified prior to analysis.

### Data Collection

This study included 45 healthy adult participants from Leuven, Belgium [24]. The average age of the participants was 24.5 (SD 3) years and ranged from 19 to 35 years. In total, 84% (38/45) of the participants were female. The participants were recruited via flyers distributed to areas around Leuven.

**Figure 1:**
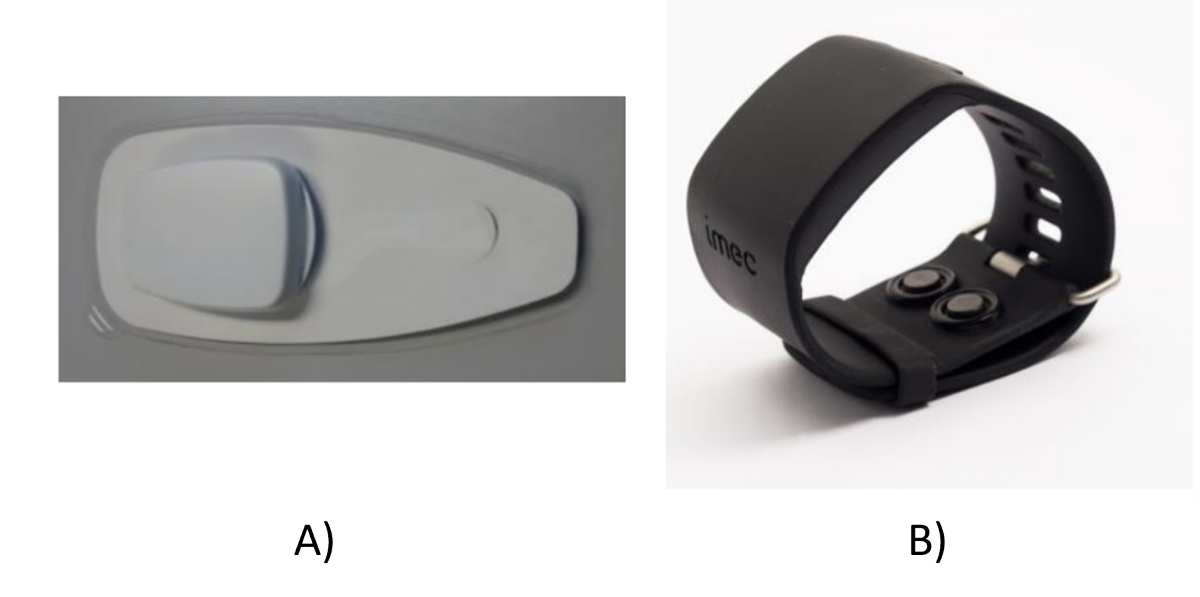
(A) Chest patch for gathering electrocardiogram and accelerometer and (B) wristband for gathering electrodermal activity, skin temperature, and accelerometer.

The study lasted for 10 days. The participants wore a sensor suite (Figure 1), including a chest patch with 2 electrodes for gathering electrocardiography (ECG) at 256 Hz and a wristband for electrodermal activity (EDA) at 256 Hz, skin temperature at 1 Hz, and accelerometer at 32 Hz. Participants were allowed to remove the device while they slept and were asked to remove the devices while bathing or participating in rigorous activities. The sensors had a battery life that surpassed the duration of the study, and the data were recorded on the device on an SD card.

Participants were given a research phone, and 10 EMAs were sent to the participants daily at random time points between 15 and 90 minutes apart. EMAs were initiated via text messages, and the participants had a specific amount of time to respond to the survey attached to the text message before it closed. The EMAs contained a question set to assess mood [25] in 3 languages: English, Belgian, and French. In total, there were 13 questions, including 9 negative (worried, stressed, anxious, annoyed, down, restless, tense, under pressure, and ashamed) and 4 positive (relaxed, cheerful, confident, and in control) affect-related questions. The questions were prefaced with the phrase “At the moment, I feel...,” followed by a rating scale for each emotion, ranging from 1 (not at all) to 7 (very much). The participants were given €0.5 (US $0.54) for each EMA they responded to.

### EMA Analysis

Our EMA question set was scored by adding the numerical interpretation of the 9 negative responses to the inverse (1 is 7 and 7 is 1) of the positive questions. The range of possible scores was between 13 and 91, with higher scores indicating more negative emotions. Owing to the low variance in reported positive and NA, we used a composite score of both positive and NA.

We also analyzed the participants’ response time (time between the notification and onset of EMA) and the response rate to EMA. We then investigated the potential for loss of engagement over time, which may lead to reduced participant receptivity. The lack of engagement may impede our capacity to discern the underlying causes of nonresponsiveness, particularly when assessing the relationship between affect and receptivity.

### EMA Receptivity and Affect Detection Models

#### Overview

**Figure 2:**
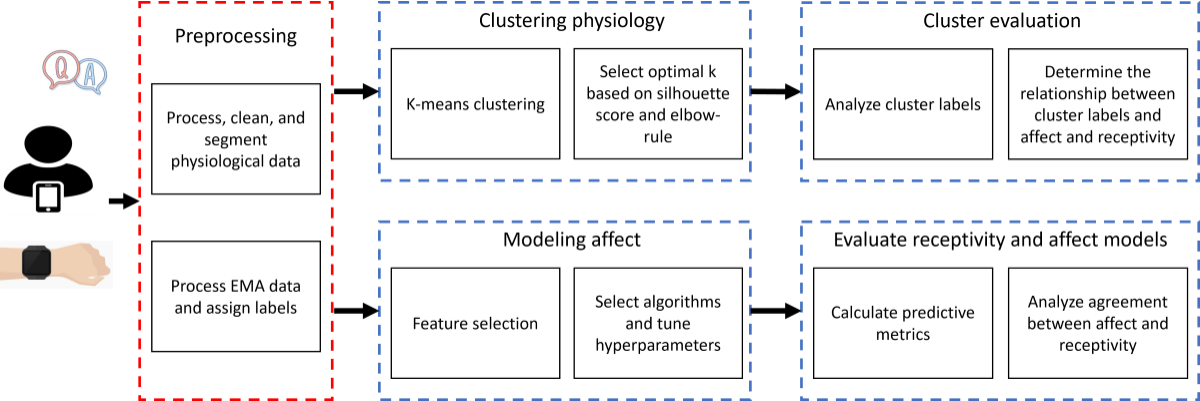
Methodology used from the raw signals to our evaluation of the relationship between affect and receptivity. EMA: ecological momentary assessment.

In the following sections, we discuss the sequential methodology, which encompasses the collection of raw signal data, the subsequent data processing and feature extraction, and the design of ML models for inferring 2 constructs—receptivity and affect. This framework is shown in Figure 2.

We began by processing our 4 sets of time series data: skin temperature, ECG, EDA, and accelerometer. Once we processed the data, we segmented them and attached labels to each segment based on the conditions explained in the *EMA Receptivity Labels* section. Next, we built and tested multiple ML algorithms to infer EMA receptivity and affect and verified the results using several statistical techniques.

#### Preprocessing

##### Time Series Processing

We began by extracting all the data from the 4 time series data sets. Table 1 shows the features computed for the 4 sets of the data. We used IQR to process skin temperature to remove outliers. We used *biosppy* [26] for the ECG to process the data and extract the R peaks. *Biosppy* uses a bandpass filter with frequencies of 3 Hz and 45 Hz, a sampling rate of 256, and the Hamilton segmentation algorithm to extract R peaks. We then validated the R peaks using an algorithm by Hovsepian et al [27]; this algorithm uses the criterion beat difference based on the maximum expected difference for a beat and the minimal artifact difference. We then used heart rate variability analysis to extract heart rate and heart rate variability features such as number of pairs of successive normal-to-normal intervals that differ by more than 20 ms and root mean square of successive differences between normal heartbeats [28]. We also obtained some frequency- and geometric-based features. For EDA, we used the method proposed by Taylor et al [29] to process and extract the statistical and wavelet features. Finally, for accelerometer, we smoothed the signal by using a fourth-order 10-Hz low-pass Butterworth filter and obtaining an average, and then, we used a package from the study by Simon [30] to extract step features. The features we extracted and the information on how those features were calculated are shown in Table 1.

**Table 1:** Features from our 3 raw sources and definitions of the features that are less commonly used.

Signal	Features	Description	Prior work
ST^a^	Mean, median, mode, minimum, range, root mean square, zero cross, kurtosis, skew, and IQR (25th percentile and 75th percentile)	Zero cross here is based on the number of times ST crosses over the mean ST. Kurtosis measures the extremity of the data in the segment, and skew is the measure of asymmetry.	[31]
ECG^b^	Mean, median, mode, minimum, range, root mean square, zero cross, kurtosis, skew, IQR (25th percentile and 75th percentile), RMSSD^c^, CVSD^d^, CVNNI^e^ SDNN^f^, NNI50^g^, NNI20^h^, PNNI50^i^, PNNI20^j^, LF^k^, VLF^l^, HF^m^, high/low-frequency ratio	Normal to Normal or RR^n^ interval indicates time between heartbeats. NNI20 or NNI50 refers to the number of successive intervals that differ by more than 20 or 50 ms. “P” indicates the proportion of NNI20 or NNI50 in the segment. RMSSD is the root mean square of successive differences between heartbeats. CVNNI and CVSD are the coefficients of variation SDNN/mean and RMSSD/mean, respectively. Our frequency domain features are based on how much of the signal lies between 0.003 and 0.04 Hz (VLF), 0.04 and 0.15 Hz (LF), and 0.15 and 0.40 Hz (HF).	[32-35]
Electrodermal activity	*Wavelet:* maximum, mean, SD, median, and above zero (1-second and half-second wavelet); *raw*: amplitude, maximum, minimum, and mean; and *filtered*: amplitude, maximum, minimum, and average	A 1-second and a half-second window were used for wavelet features. Features were calculated for both the first and second derivatives of each window size.	[31,36-39]

^a^ST: skin temperature.

^b^ECG: electrocardiography.

^c^RMSSD: root mean square of successive differences between normal heartbeats.

^d^CVSD: coefficient of variation of differences between adjacent normal-to-normal intervals.

^e^CVNNI: coefficient of variation of the normal-to-normal intervals.

^f^SDNN: SD of the normal-to-normal intervals.

^g^NNI50: number of pairs of adjacent normal-to-normal intervals differing by more than 50 ms.

^h^NNI20: number of pairs of adjacent normal-to-normal intervals differing by more than 20 ms.

^i^PNNI50: percentage of pairs of adjacent normal-to-normal intervals differing by more than 50 ms.

^j^PNNI20: percentage of pairs of adjacent normal-to-normal intervals differing by more than 20 ms.

^k^LF: low frequency.

^l^VLF: very low frequency.

^m^HF: high frequency.

^n^RR: R-peak to R-peak.

##### Segmentation

We segmented the data into 1-minute windows with a 30-second overlap. We then calculated the statistical features for each of the sensors, excluding steps. For each of these windows, we calculated historic features. To do so, we elongated each of the windows by 5, 30, and 60 minutes and then extracted the features with the extended window size (ie, for each 1-min window, we have not only the features from the 1 min but also the features going back to these 4 time frames).

##### EMA Receptivity Labels

**Figure 3:**
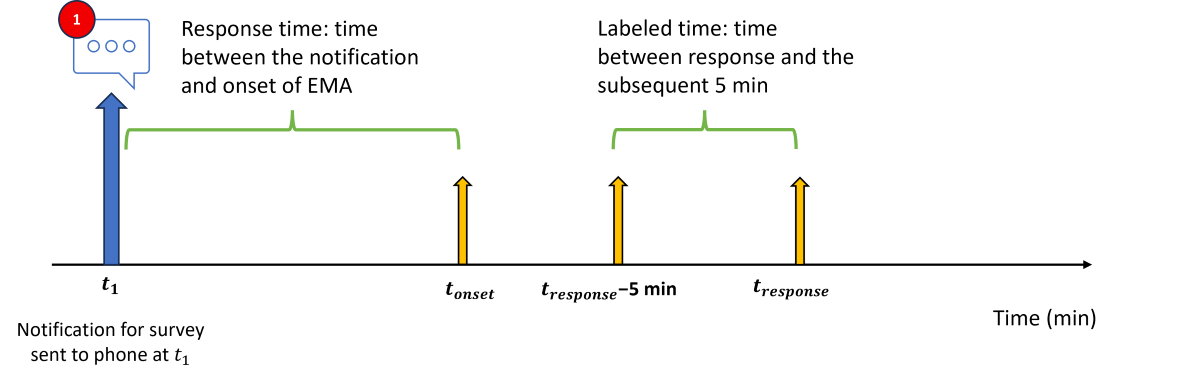
Representation of our labeled segments for the 5-minute window. It also demonstrates how we calculate response time (time between notification [t1]) and the start of the ecological momentary assessment (EMA; tonset).

Labels for receptivity were based on whether the user responded to the EMA and were assigned to segments based on whether it was within a specified time of the scheduled notification. By expanding the window of labeled data, we can increase the size of the labeled data set (pseudolabeling). However, as this window increases, so does the distance between some of our time points and the corresponding label. We tested windows that are 5, 30, 60, and 120 minutes long. For instance, for the 5-minute window, if an EMA was sent at midnight, the segments that fell between 11:55 AM and midnight would be labeled “responded” if they did respond and “no response” if they did not. We applied the same method for the affect labels (Figure 3). We ultimately chose 30-minute windows owing to the balance between the size of the training set and the labeled points being relatively close in terms of time to the actual response (or nonresponse).

##### Affect Labels

Previous research on monitoring and tracking emotional states using wearables has commonly used binary or categorical affect measures to detect emotions [40]. These psychological instruments often feature well-defined categorical score representations, which make it easier to distinguish between emotions. The distribution of the reported composite affect scores made defining an adequate categorization of the labels difficult. Most participants reported positive emotional states, which complicated setting an appropriate cutoff. Setting the cutoff at a high value would result in an imbalanced set of labels, whereas selecting a lower value would create a balanced data set but lack logical consistency. For instance, choosing a cutoff of 26 to distinguish between positive and negative emotions would lead to a balanced data set. However, the range of possible responses was between 13 and 91, so a response would be considered negative even if the participant indicated relatively positive or neutral emotions.

In response to these challenges, we used recorded composite affect values as our class labels and designed our ML algorithms as a regression problem. Although this method of affect inference is less commonly found in the literature, it prevents the need for arbitrary data classification. Given that the data exhibited an inherent imbalance, with less frequent occurrences of negative emotional states, using regression may still affect our ability to predict these less common negative emotions.

#### Analysis of the Relationship Between Features and Receptivity and Affect

We examined the significance of each feature in terms of its ability to predict affect and receptivity. To do so, we conducted a repeated measures ANOVA test to assess how well each feature is related to the response class labels. In addition, we used a linear mixed model (LMM) to investigate the relationship between features and affect scores. We used an LMM because we worked with constant labels instead of converting the affect score into binary or categorical values, as done for receptivity. The dependent variable (affect score) was regressed on the fixed effect variable (features), while accounting for random effects (participant ID). These tests help identify any features or signals that may have significance in determining receptivity or affect.

#### Receptivity and Affect Model Design and Hyperparameter Tuning

We designed ML models to infer EMA receptivity and affect. A wide variety of ML algorithms are used in affect and receptivity prediction including random forest (RF) [31,32,39], support vector machine [33,34,39], logistic regression, *k*-nearest neighbors [30], neural network (NN; long short-term memory, recurrent NN, convolutional NN, etc) [31,39], and naive Bayes [39,41]. On the basis of our sensor data, initial tests, and drawing inspiration from previous studies, especially those by Mishra et al [10,11]. We selected (1) RF for predicting emotional state and receptivity, (2) an NN for predicting emotional state, and (3) a baseline model. This baseline model serves as a benchmark for evaluating whether our models outperform random chance, whereas the NN algorithm was introduced as a possible improvement on existing model implementations. Unlike the research mentioned previously, we used physiological data rather than contextual data. These signals are sampled at higher frequencies compared with contextual data and allow the extraction of more fine-grained features, making NNs more feasible. We designed personalized models to infer the receptivity and effect of EMA.

To optimize our personalized model, we selected hyperparameters using the grid search method for each participant, explicitly using the *GridSearchCV* method defined in *scikit-learn*. This method uses an exhaustive search method (ie, testing each user-defined parameter permutation). The hyperparameters tested included the number of estimators, maximum depth of the estimator, minimum number of samples per leaf, minimum number of samples for split, and maximum number of features that can be used for the split. Using training and validation sets, we selected the parameters and then applied the optimal model to our test set. The optimal set of hyperparameters differed for each participant, although the most common optimal hyperparameters chosen included 60 estimators, maximum depth=3, minimum sample leaf=2, minimum sample split=2, and maximum features=square root of the number of features.

Our NN model was structured to use 3 densely connected layers using a rectified linear unit activation function at each layer. The output dimension of each layer was 256, 128, and 64, and the output layer was a densely connected layer with 2 output dimensions. The reasoning for an output layer of 2 is to define a CI for our regression model.

The baseline model was built by predicting random output based on the distribution of the class labels in the training set (ie, if 10/100, 10% of the labels were nonresponses and 90/100, 90% were responses, the model would predict nonresponses 10/100, 10% of the time). We can determine the expected outputs for this model; our true positive rate should be equal to *Pr(response in the training set) × Pr(response in the test set)*. The more evenly the class labels are distributed, the worse the performance of the model. For the affect regression models, we used a normal sampling method with the mean and SD based on the training set class labels.

As there are more labeled responses compared with nonresponses, we considered this imbalance in the receptivity prediction model, weighting the classes based on the distribution in our training set. All models were built using the Python packages *scikit-learn* [42] or *Tensorflow* [43].

#### Model Uncertainty

To determine the relationship between affect and receptivity, we must use predictions to infer the emotional state of our participants during nonresponses. As affect is a complex and difficult-to-predict construct, we need a method for filtering our predictions based on some level of confidence. Therefore, we introduced a method for calculating uncertainty for regression using an NN.

Determining a confidence value for a regression model is difficult compared with a binary or categorical model. We can use a custom loss function in our NN to estimate epistemic and aleatoric uncertainty for our regression model, where epistemic uncertainty is based on our ability to predict our class labels with the available data (affected by lack of knowledge or data), and the aleatoric uncertainty is affected by randomness, which is unknown or unmeasured in the model [44].

Our affect prediction model outputs are 2D rather than a single predicted output. The first output is the predicted affect, µ(x), and the second output, ln(σ(x)), is the predicted variance (the log allows us to take the exponent to ensure a positive value for σ). Both µ and σ are functions of our training set x.



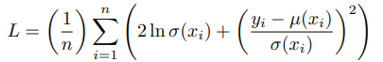



The loss function L is shown in the equation (custom loss function for measuring model uncertainty) and is derived from the mean square error (MSE) calculation and the maximum likelihood of a normal Gaussian distribution [45]. The numerator of this equation is identical to the MSE loss function, where µ(x) is the predicted output of our model. Unlike the MSE loss function, we continuously update not only our predicted output µ but also the predicted variance σ. The σ output of our model is based on error; the sigma value increases to account for higher error and decreases to account for lower error. This σ value can be used as an uncertainty or error metric. Although it is still a predicted value, it should align with how confident the model is in the σ(x) output. The σ value plays a crucial role in assessing the confidence of our affect predictions, given that we use predicted affect to infer emotional states during nonresponses.

Consequently, to illustrate the relationship between the predicted sigma value and model uncertainty, we performed a mixed effect model analysis using affect scores and the predicted sigma values and tested whether greater uncertainty will occur in emotional states that are less frequently represented and when the testing error is larger. As uncertainty is a measure of the model’s confidence in its predictions, we can reasonably assume that predictions associated with larger testing errors would correspond to higher levels of uncertainty.

#### Model Evaluation

For cross-validation, we used a personalized random train-test split cross-validation method. We randomly split the data into training and testing sets using the response label (whether they responded to the EMA or not) to stratify the split. Responses and nonresponses can encompass multiple segments; by grouping them together, we avoid splitting up segments from a single response or nonresponse. As our response labels are unbalanced, we want to ensure that our training, validation, and test sets have a relatively even number of responses and nonresponses. For the purpose of fairness, we excluded 3 participants who had a single nonresponse from our receptivity results.

We first normalized the training and test sets independently of one another based on the participant. In total, we obtained approximately 230 features from the sensor signals. We reduced our feature set using principal component analysis. Our implemented principal component analysis was set such that the number of produced components explained 99% of the variance (48 features). This method was used for each model, excluding the RF model, in which the original normalized data were used as the input.

### Analysis of the Relationship Between Affect and Receptivity

#### Overview

We conducted two different analyses to understand the relationship between affect and receptivity better:

1. To infer emotional state during nonresponses, we clustered the physiological data and then examined the makeup of the clusters. By doing so, we can assume the emotional state of different clusters and unlabeled data points.
2. For EMAs the participants did not respond to, we used the affect prediction model described in the previous section to infer the emotional state at the time of a nonresponse. With these newly predicted affect scores, we can analyze the differences in the emotional state during a response and nonresponse.

#### Cluster Evaluation

We used the most significant features (based on correlation) when predicting receptivity for our clustering analysis. To determine the optimal clustering method, we tested several clustering methods, including hierarchical and *k*-means clustering, with a maximum number of iterations of 300. We then calculated the silhouette score across all clusters using receptivity as our ground truth and selected our best-performing set of hyperparameters. On the basis of the cluster distribution, we analyzed the difference in the perceived emotional state of the participants. We calculated the average NA, PA, and receptivity rates in the clusters for each participant and then characterized the clusters based on receptivity rates (high receptive and low receptive clusters). Next, using repeated measure ANOVA, we demonstrated the statistical difference between affect and the clusters. Given that the clusters were created from physiological data, we know that the data points within each cluster are physiologically similar; therefore, we inferred that they would also exhibit similar psychological states. This allowed us to assign affect scores to nonresponsive data points within each cluster based on the labeled data points within that cluster. Unlike affect prediction, we used the raw NA and PA values in our evaluation as the clustering was performed independently of affect scores; therefore, the lack of variance in responses did not affect the output of the clustering. These results gave us a sense of participants’ perceived emotional state during nonresponses. We also investigated differences in receptivity in 2 clusters using the chi-square test.

#### Analysis of the Receptivity and Affect Relationship

Ideally, we would show the interaction between affect and receptivity using the data collected. However, because nonresponses do not have a corresponding affect score, we designed and implemented our models for receptivity and emotional state.

After generating predictions for our test data set, we assessed the agreement (using Cohen κ) and correlation (using the point biserial method) between receptivity and predicted affect, leveraging true labels at time points when affect measures were reported. A high level of agreement or correlation would suggest a strong relationship between these 2 constructs, thereby highlighting the potential influence each construct would have on an ML algorithm to predict the other construct. We then examined the disparities between the predicted affect during nonresponses and the reported affect during responses. By doing so, we can establish the extent to which emotional state influences receptivity. Substantial disparities in affect between responses and nonresponses suggest that participants’ emotional states impact their receptivity. Consequently, a model designed to predict receptivity would indirectly include emotional state as a determinant of a participant’s receptiveness. However, it is essential to acknowledge that some of these variations could be attributed to model error. As a result, we also compared the predicted and reported affects during responses to investigate the significance of the model error. We then calculated and visualized the cumulative distribution of these 3 sets of values to illustrate the influence of affect on receptivity and the associated model error.

Finally, we investigated the potential effects that an ML-based receptivity algorithm would have on reported affect, influencing the outcome of the study. On the basis of our receptivity model, we can estimate the difference in the reported perceived emotional state between our true findings and predicted affect during time points that would initiate an EMA.

## Results

In the following sections, we discuss the results of our study, particularly the methods of evaluation that were discussed in the previous section.

### EMA Analysis: Affect and Receptivity

**Figure 4:**
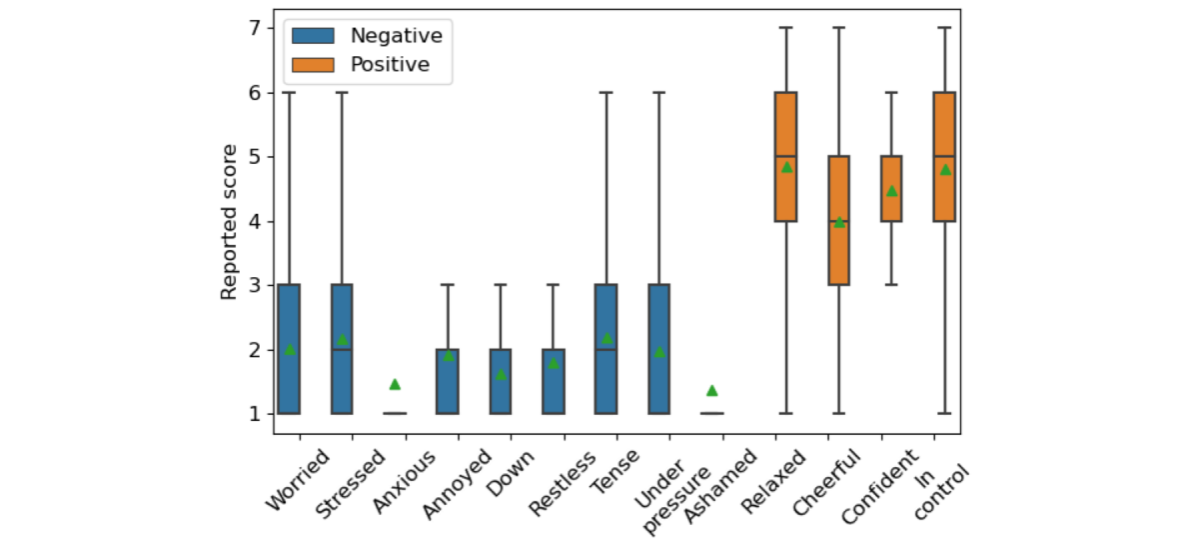
Question set: includes the 13 questions used to measure affect with their mean, SD, and correlation to the final affect score. For each question, participants were asked to rate the degree they were experiencing each emotion. These 13 questions can be split into positive affect (orange) and negative affect (blue).

**Figure 5:**
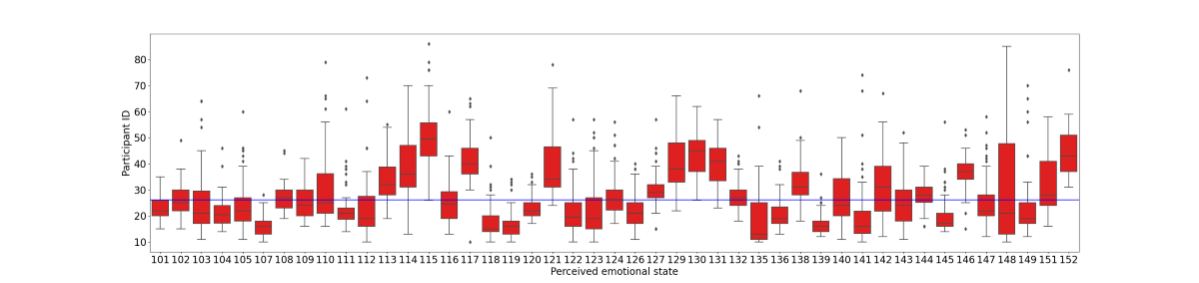
Box plot of perceived emotional state, minimum is 13 (negative) and maximum is 91 (positive). The average perceived emotional state is 26.42, denoted by the blue horizontal line.

The distribution of EMA responses is shown in Figure 4. Although participants rarely indicated high negative emotions, this trend is evident in Figure 5, illustrating a box plot of composite scores for each participant. Participants’ average and median reported affect were <26, meaning that, on average, the participant responded to each question with a relatively low score of 2 (on a scale between 1 and 7, where 1 indicates high positive emotion and 7 shows high negative emotion). We also investigated participants’ emotional states as the study advanced and observed minimal to no variations based on their duration of enrollment or time of day.

On average, participants responded with a 4.5 for PA questions and a 1.8 for NA questions. This disparity in affect intensity was consistent with previous research [23]. There was a slight difference in the reported affect between male and female participants. On average, female participants responded with a 1.9 (SD 1.08) for the NA questions and 4.5 (SD 1.3) for the PA questions, whereas male participants responded with a 1.8 (SD 0.9) for the NA questions and 4.7 (SD 1.0) for the PA questions.

Of the 3885 notifications sent to the 45 participants, there were 3066 (78.92%) responses. As the study persisted, there was little to no drop-off in receptivity rates over time. This finding helped confirm that loss of engagement was not a contributing factor to receptivity. Most studies have stated that the quality receptivity rate is at 80%. The range of response time (time between notification and initiation of the EMA; Figure 3) was between 0.5 seconds and 306 seconds. Participants responded to the notification on average in 20.9 seconds and had a median response time of 8.7 seconds. There were no responses after 306 seconds of a notification. The reason for this fast response time is that participants were allowed 90 seconds to begin the survey, after which the survey would no longer be accessible (we had a few responses after the 90-second restriction owing to software or design issues). This restriction makes it challenging to relate response times to participant affect, as has been done by other researchers.

We found that none of the mood responses were strongly correlated with the time to respond. Across each question, we did not obtain a correlation coefficient >0.03 (all correlations indicated significant confidence; *P*<.05). This low correlation coefficient indicates that the participant’s mood had little to do with how long it took the participant to initiate the EMA. Although considering the limit we put on the response time, this relationship might be difficult to assume.

### Analysis of Features

The features that we found to be the most significantly related to receptivity were ECG low frequency (1 min, momentary: *F*_2,54_=6.7; *P*<.001) and very low–frequency features (1 min, momentary: *F*_2,54_=4.7; *P*=.02 and 60 min: *F*_2,54_=4.1; *P*<.001); EDA mean (*F*_2,54_=10.2; *P*<.001) and median (*F*_2,54_=15.4; *P*<.001); number of pairs of adjacent NN intervals differing by more than 20 ms in the 5 and 60 minute windows; percentage of pairs of adjacent NN intervals differing by more than 50 ms in the 30 minute window (*F*_2,54_=11.2; *P*<.001); and maximum (*F*_2,54_=6.3; *P*<.001), minimum (*F*_2,54_=3.6; *P*=.009), and absolute maximum (*F*_2,54_=6.6; *P*=.002) of the first and second derivatives for EDA. These results show that ECG and EDA-related features were best at differentiating between responses and nonresponses compared with features derived from accelerometer and skin temperature.

When running the LMMs to determine the relationship between features and emotional state, we found a nonsignificant relationship between affect scores and steps or sleep features. However, heart rate was significant when predicting emotional state, particularly negative emotion. This LMM showed a significant positive relationship between heart rate and affect (β=.007; *P*<.04). This underscores the significance of heart rate as a predictor of emotional state, although it does not necessarily imply that steps and sleep features lack importance in this context.

### Receptivity and Affect Models

After processing, cleaning, and filtering out segments with confounding values, we obtained 1368 responses with usable physiological data. As our class labels were expanded to include segments 30 minutes before the point of response (pseudo labeling), we ended up with 13,477 data points for determining affect and 17,254 data points for predicting response.

#### Model Performance

Table 2 shows the results of our receptivity (binary) and affect (regression) models. On the basis of these results, there was little difference between the RF and NN models, although we used the NN models to demonstrate the relationship between affect and receptivity in the following section.

**Table 2:** Model results for predicting receptivity (binary) and affect (regression).

Model	Accuracy, mean (SD)	Precision, mean (SD)	Recall, mean (SD)	*F*_1_-score, mean (SD)	Root mean square error (SD)
Baseline	0.73 (0.001)	0.83 (0.002)	0.84 (0.002)	0.83 (0.002)	11.1 (4.3)
Neural network	0.84 (0.19)	0.82 (0.006)	0.85 (0.10)	0.86 (0.20)	7.3 (2.7)
Random forest	0.83 (0.11)	0.82 (0.15)	0.94 (0.10)	0.87 (0.12)	7.5 (3.1)

#### Analyzing Uncertainty in the Affect Model

**Figure 6:**
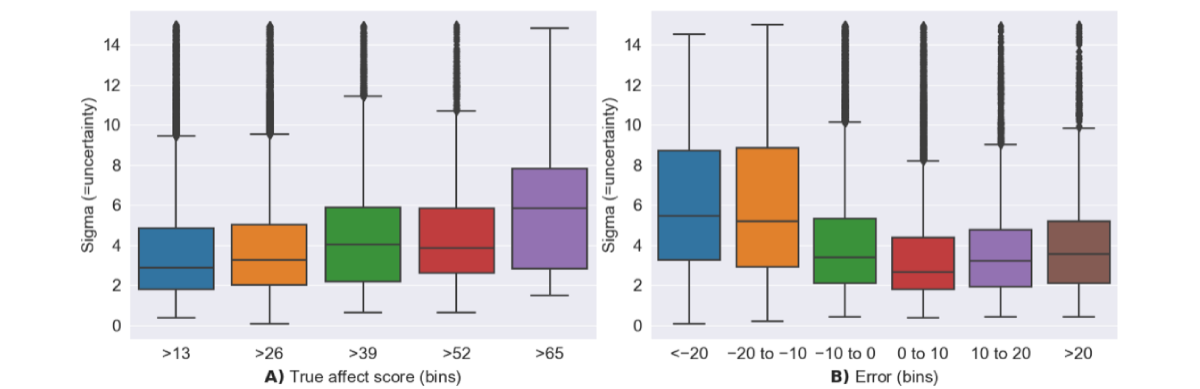
Box plot showing the σ value for (A) true labels and (B) predicted error.

Figure 6A shows the relationship between the calculated sigma value (uncertainty) and the reported affect scores. Uncertainty should follow a pattern where class labels that are more represented in the training set should have lower uncertainty. Conversely, values that are less represented in the data set should have larger uncertainty. As can be seen, Figure 6A σ values are smaller when the reported emotional state is more positive. As shown in Figure 5, most respondents indicated relatively low composite scores, with a few participants reporting an affect score >40. We also observed a statistically significant relationship between sigma and affect scores, as shown in Figure 6A, using a mixed effect model. In this model, we accounted for the random effect associated with participants, as indicated by the mixed linear model results (intercept: 7.090; *P*<.001 and affect score: 0.002; *P*=.046).

Figure 6B shows the relationship between σ and the testing error; in particular, σ values were larger when the model was further from the ground truth. This relationship shows that our σ value is an accurate representation of model uncertainty. On the basis of Figure 6, we can say that the σ value we calculated is related in some way to uncertainty. Figure 6B shows that most responses indicating an affect score of <39 had a σ of <6. Therefore, we chose 6 as the cutoff for uncertainty. This cutoff filters out many of the predictions that are more likely to have higher errors because we cannot look at errors during nonresponses, as we have no affect label.

### Receptivity and Affect Analyses

#### Cluster Analysis

On the basis of the “elbow rule” of silhouette scores, we chose *k*-means as our clustering method with 2 clusters. We found that the distribution of receptivity was somewhat different between clusters. Cluster 0 contained a higher density of responses, with just <15% nonresponses, whereas cluster 1 had a higher density of nonresponses of just >21%. We first analyzed the overall affect scores in the 2 clusters, where we found the average reported affect score in cluster 1 to be >3 points higher than the average reported affect in cluster 0 (repeated measure ANOVA, *F*_2_=23.16; *P*<.001). The receptivity rates and average reported affect scores for the 2 clusters are shown in Table 3. We also found that the distribution of receptivity was different between the 2 clusters using the chi-square test of independence (χ^2^_2_=898.8; *P*<.001). These results indicate distinctions between response and affect across the cluster labels. Considering that the cluster with a higher density of nonresponses (cluster 1) also had a higher average affect score (higher scores indicate more intense negative emotions or lower positive emotions), we can assume that there was a relationship between EMA receptivity and reported affect.

**Figure 7:**
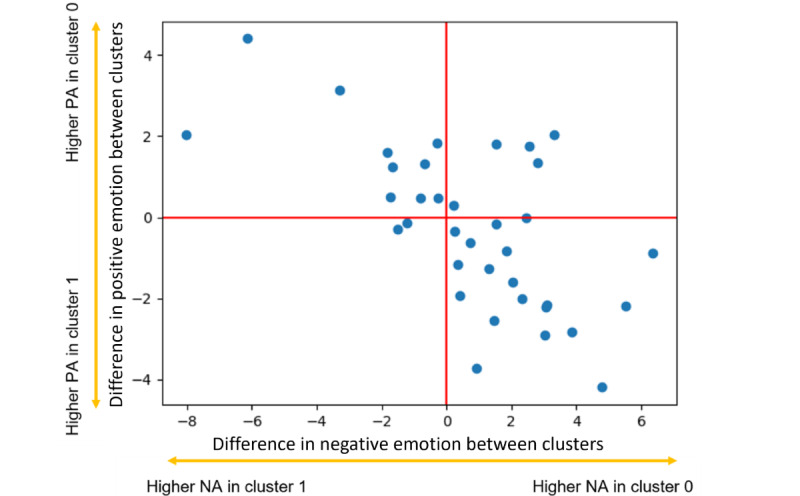
Each point represents a participant, where the x-axis denotes the difference between average negative affect (NA) of cluster 0 and 1, whereas the y-axis represents the difference between average positive affect (PA) of clusters 0 and 1.

Figure 7 shows a scatter plot of the difference in perceived PA between the 2 clusters and the difference in perceived NA between the 2 clusters for each participant. The results show that participants’ perceived emotion was more negative regarding lower PA and higher NA in cluster 1 compared with their perceived emotional state in cluster 0. As stated earlier, cluster 1 contains a higher percentage of nonresponses compared with cluster 0, indicating that cluster 1 is a better representation of a nonresponse. Therefore, it appears that there is a relationship between negative perceived emotional state and receptivity. Using the cluster labels as groups, we calculated the *F* test statistic using an ANOVA test for each feature. The features that separated the 2 clusters were mostly calculated from the ECG signal, including the minimum heart rate, low or very low frequency, mean heart rate, coefficient of variation of the NN intervals, coefficient of variation of differences between adjacent NN intervals, high frequency, and maximum heart rate (in order of *F*_1_-score). Features obtained from the EDA, accelerometer, and body temperature did not return significant *P* values when calculating the *F* test statistic.

**Table 3:** Receptivity rates and average reported affect scores in each cluster.

Cluster number	Receptivity rate	Reported affect score, mean (SD)
Cluster 0	0.85	24.3 (4.7)
Cluster 1	0.78	27.3 (4.9)

#### Relationship and Analysis Between Receptivity and Affect

**Figure 8:**
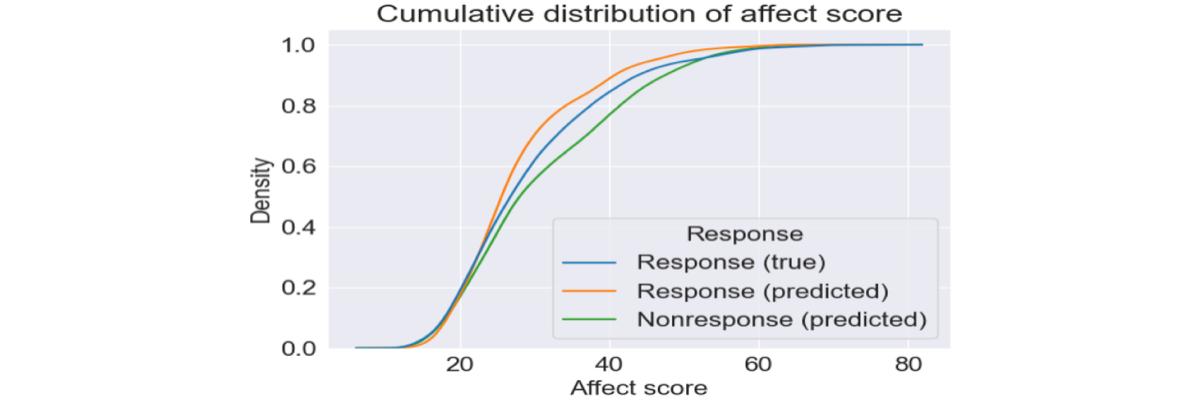
Cumulative distribution of predicted and actual affect scores for responses and nonresponses.

Figure 8 shows the cumulative distribution of reported affect scores for responses and predicted affect scores for responses and nonresponses. On the basis of this figure, there is a clear difference between the predicted affect during nonresponses and our true affect scores. Although this could be a model error, we also predicted affect scores during these responses and found that our model consistently predicted lower affect values (higher PA).

There was a fair amount of agreement between our affect and our binary response model, with a Cohen κ score of 0.33 and a correlation of 0.44. When our model predicted a response, 77.42% (22,761/29,399) of the segments were during times when the affect model predicted PA. Only 69.72% (7760/11,131) of the predicted nonresponses reported PA. This indicates that the predicted response is negatively related to affect (ie, responses are associated with PA, whereas nonresponses are associated with NA). The reason determining the relationship between our constructs is important is because this bias can, and as we show, affect the overall outcome of a study. For instance, the average predicted affect score for times that we predicted as low likelihood for a response was a full 1.5 (SD 1.35) or 2.01 points higher than the average predicted affect for points predicted to be of high likelihood for a response. When observing only the segments where we misclassified a response (ie, we had a true affect, but the response was misclassified as a nonresponse), we found that the average affect score dipped slightly from 26.1 (predicted nonresponse) to 25 (predicted response). This difference in affect between responses and nonresponses is evidence that our receptivity model is indirectly based on affect. The SD of the affect score also decreased from 11.1 (true labels) to 9.8 (true affect and predicted response) during responses.

**Figure 9:**
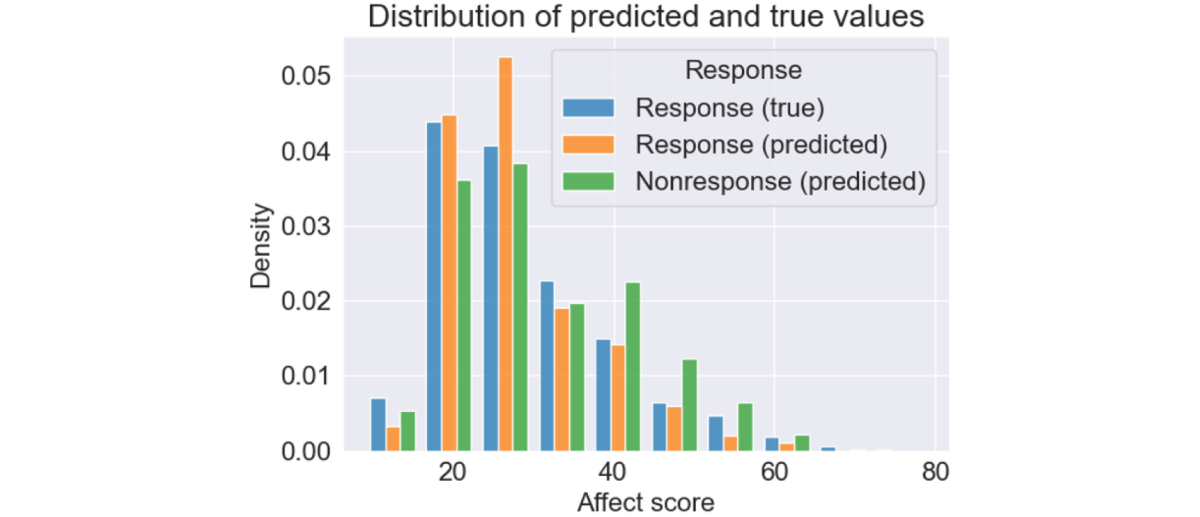
Distribution of predicted and true affect scores for responses and nonresponses. Density is specific to the response and nonresponse.

The average predicted affect score for a nonresponse was 30.9 (SD 11.2), and the average affect score for a response was 29.3 (SD 10.7; true) and 27.7 (SD 8.9; predicted). The predicted affect scores during nonresponses were higher than the reported and predicted affect scores during responses. Given that our average testing error was −1.6, we could also assume that the predicted affect during these nonresponses could be more negative than the true predictions. The distribution of these scores is shown in Figure 9. In Figure 9, all 3 groups’ affect scores peaked at around 20 to 25; this is probably owing to the large number of reported affect scores in this range. However, nonresponses had a second peak at an affect score of 40. This bimodal distribution could indicate that our affect distribution during nonresponse was affected by ≥2 factors. Some nonresponses may not be affected by their affect but perhaps by their daily life activities (seeing a movie, spending time with family, showering, etc). In contrast, the second peak indicates that NA is related to nonresponses.

## Discussion

In this section, we discuss the outcome of our study, particularly the relationship between emotional state and receptivity, what that means, how it affects our results, and how we might implement a receptivity model that removes this bias. We also mention the limitations of this study.

### Principal Findings

This study aimed to understand how ML models used to improve participant receptivity can affect the outcome of a study. Although we focus on emotional state in this study, we feel as if there are many health constructs and outcomes that can be affected by these receptivity models. Improving receptivity is not a new concept, but in the realm of mHealth, it is an emerging problem. The factors influencing study adherence have been analyzed and discussed in depth in previous research. One such scope is in medication adherence. Researchers have found many factors that influence medication adherence, from social, therapeutic, patient-related, and disease-related factors [46]. However, few have examined the momentary factors that affect adherence to medication or a health construct, and few have had the ability to do so without wearable sensors and momentary assessments.

Our findings using supervised learning and clustering indicate a clear relationship between emotional state and user receptivity. The clustering method demonstrated clear differences in affect between a highly receptive cluster and a less receptive cluster. The results of supervised learning demonstrate that users experience more negative emotions during nonreceptive time points. Although our results showed promise for a model dedicated to predicting response, we also showed the biases inherent in such a model. Ideally, we would want a receptivity model that is completely independent of emotion. Otherwise, we are influencing the participant’s responses.

Our results demonstrate that an mHealth study implementing a receptivity trigger based purely on the likelihood of responding (a model that triggers EMAs and JITIs using predicted receptivity) will bias the participant’s response. In this case, the model would initiate an EMA or JITI during times of more positive emotions, thereby decreasing the overall affect score for the EMA and possibly sending the JITI during times when the intended construct was not being met. As our ability to predict binary affect is limited with this data set, we believe that using the affect regression and ground truth labels for responses will return the most realistic representation of affect during nonresponses.

### Comparison With Prior Work

Our findings are consistent with those of the previous studies. Many prompt-level studies [21-23] found a relationship between nonresponses and higher levels of NA in previous prompts. Although these results can provide insight into what makes a participant less compliant with EMAs, they do not offer a reasonable method for using this information in real-time decision-making. Using ML, wearable sensors, and contextual cues allows researchers to predict noncompliance components and distribute EMAs accordingly.

Consistently, our models either surpassed or achieved equivalent performance compared with previous research efforts. We achieved F_1_-scores ranging from 0.83 to 0.87 when predicting receptivity. In contrast, Künzler et al [14] reported F_1_-scores of approximately 0.4 while relying solely on contextual features. It is important to note that these results are not directly comparable, as contextual data lack the granularity of the data collected in our study.

Regarding affect prediction, our results present a unique challenge for comparison because we used regression in our predictions, unlike most researchers who typically use binary or categorical labels for emotion recognition. We chose not to convert our ground truth data into binary or categorical labels because of the inherent ambiguity in setting the thresholds and the limited variance in user responses. The effectiveness of affect prediction can vary significantly depending on the specific construct of interest and the sensors and signals available. Schmidt et al [40] conducted a review and reported an emotion recognition accuracy ranging from 40% to 95% using wearable sensors and signals. In terms of regression analysis, Tuarob et al [47] achieved nearly identical root MSE scores (PA=7.37; NA=7.40) when forecasting positive and NA scores from Positive and Negative Affect Scale using RF regression and previously collected questionnaire data.

### Limitations

In this section, we address the limitations of our study, which can be categorized as limitations in our population, study design, data collection, and affect prediction models.

The major concern of our study population is that our results may be specific to this cohort. The study population was very receptive, even with 10 EMAs sent daily. This could be difficult for other researchers to implement, as the frequency and complexity of the EMA are fairly burdensome. Although we believe that the relationship between affect and receptivity would extend to other studies, it is important to note that our population was relatively small (N=45), predominately young (age 24.5 y), and had a higher representation of female participants (38/45, 84%). Consequently, our results may be specific to our cohort and EMA question set, but previous studies analyzing medication adherence and prompt-level relationships between EMAs and nonresponses indicate that the effect of emotional state on receptivity is common across multiple populations [21-23]. Further research is needed to explore the extent of this relationship between different emotional states and receptivity across multiple populations.

One limitation of the study design is that we cannot examine how loss of engagement over time affects the relationship between emotional state and receptivity. There was little to no drop-off in receptivity rates as our study progressed. This may have been because of the relatively short time frame in which the participants were enrolled. As a result, it is difficult to explore the effect emotional state would have on EMAs in the latter part of a study when participants can be more fatigued and less engaged. In future work, we intend to study a population for an extended period to analyze how emotional state affects participant response rates later in the study. Ideally, this will allow us to see the rate at which responses decay, the causes, and how we might combat it. Furthermore, we believe that a measure of this decay in engagement could be included in our ML-based decision-making for delivering EMAs that mitigate study fatigue, similar to how we would use model uncertainty to diversify emotional response.

Another potential study design limitation is that the app and research phone were shared with participants. Carrying 2 phones, especially one dedicated solely to responding to EMAs, can be burdensome for participants. In addition, the app designed for EMA distribution requires further usability evaluation. In future work, we aim to develop an app that can seamlessly integrate into users’ devices and assess its ease of use.

The data gathered in this study were limited to physiological features and user-defined responses. Although the physiological features make up a large portion of what researchers consider important for predicting psychological constructs, the data set lacks sampling contextual data. Certain contextual information is imperative for recognizing emotions and improving EMA response rates that cannot be obtained using physiology, such as social context. The social context can help infer the participant’s emotional state and willingness to respond to an EMA or JITAI.

Similarly, by incorporating more psychological and environmental cues (personality traits, working hours, etc), we can better understand what to expect from our participants regarding receptivity and affect before the start of the study. Using these prestudy measurements, we could assess the type of participants enrolled. Specifically, what will be their needs regarding receiving and responding to EMAs. This will help us develop and personalize our ML models for affect and receptivity.

The last significant limitation of our study is the use of predicted affect labels in determining the relationship between emotional state and receptivity. We can never collect reported affect during nonresponses for this or any data set. We attempt to reduce this limitation by using uncertainty to filter out less-confident predictions. Nevertheless, the predicted affect is only as good as our models. The only way to overcome this limitation is to improve the affect models. Although some may argue that the quality of our models needs to be more robust to claim a relationship between affect and receptivity, the effects of emotional state on engagement in social and daily life activities are well documented and consistent with our conclusion.

### Conclusions

This paper presents the possibilities for bias in ML models to trigger surveys and interventions for participants in mHealth studies. Our results show a clear relationship between emotional state and user EMA receptivity. By designing an mHealth study using a “trigger” to improve participant response, it is imperative to consider some biases that may arise, in this case, affect. Participants were more likely to respond to an EMA during positive emotional states. If we distribute those EMAs to times when they are more likely to respond, we would further be biasing our participants’ recorded emotional state. Although this may not be a significant problem for less responsive populations, for the general population, this could change researchers’ perception of the participant’s perceived emotional state. In this study, we did not examine other constructs that might be a factor of receptivity because affect is the focal point of this study. For this objective, we are collecting both subjective and physiological data. Although this may be broad, it can be applied to any construct, particularly the intended construct of an mHealth study.

The pitfall of any mHealth study, particularly those involving psychological concepts, is the dependency on subjective user responses. The sampling rate of subjective responses will always be less than that of the physiological sensors and even some contextual cues. As our feature set became increasingly comprehensive, our labeled data remained relatively sparse. Considering that our proposed trigger considers factors beyond receptivity, it would likely have lower receptivity rates compared with triggers solely based on receptivity. However, the importance of even a minimal increase in a user’s adherence or engagement in a study can drastically improve researchers’ understanding of the health construct.

The models discussed in this paper have mostly proposed single-objective optimization functions that try to optimize based on whether the model considers that a user will respond to an EMA. In future work, we will propose a multiobjective optimization function for triggering EMAs and JITAIs based on the likelihood of responding and an active-learning measurement of the health construct. This multiobjective function would base the timing of the EMAs on 2 separate objectives: receptivity and model uncertainty. By initiating EMAs or JITAIs based on these 2 objectives, we can obtain an expected response that is more diverse in terms of affect. We hope that the work presented in this paper can be used to further enhance communication and the ability to gain knowledge from participants.

## Abbreviations

ECG: electrocardiography
EDA: electrodermal activity
EMA: ecological momentary assessment
JITAI: just-in-time adaptive intervention
JITI: just-in-time intervention
LMM: linear mixed model
mHealth: mobile health
ML: machine learning
MSE: mean square error
NA: negative affect
NN: neural network
PA: positive affect
RF: random forest
